# Preliminary Aspects Regarding the Anticorrosive Effect of Multi-Layered Silane–Hydroxyapatite Coatings Deposited on Titanium Grade 2 for Medical Applications

**DOI:** 10.3390/ma17236001

**Published:** 2024-12-07

**Authors:** Agata Dudek, Oliwia Kierat

**Affiliations:** Department of Material Engineering, Faculty of Production Engineering and Materials Technology, Czestochowa University of Technology, Aleja Armii Krajowej 19, 42-200 Czestochowa, Poland

**Keywords:** titanium, surface modification, sol–gel method, silane coatings, hydroxyapatite coatings

## Abstract

This paper presents a method for producing VTMS/HAp/VTMS/VTMS multilayer coatings on a Grade 2 titanium substrate and characterizes their structure and functional properties. Two solutions were used to produce the coatings: one based on vinyltrimethoxysilane (VTMS) and the other on hydroxyapatite (HAp) powder. The coatings were applied using immersion using the sol-gel method. Microstructural tests of the multilayer coatings were performed, their chemical composition was determined, and the structure was characterized using Fourier Transform Infrared Spectroscopy (FTIR). A detailed analysis of the geometric structure of the coatings was carried out both before and after corrosion tests. The geometric structure of the multilayer coatings was analyzed using a light microscope and an atomic force microscope (AFM). The thickness of the coatings was determined using a Testan DT-10 AN 120 157 m, and the adhesion of the coatings to the substrate was analyzed using Scotch™ tape. The corrosion resistance of the coatings in simulated body fluid was tested to evaluate their suitability for implantology. As demonstrated by the research presented in this paper, the sol–gel process can successfully produce silane coatings by adding hydroxyapatite powder. The new materials proposed in this study can effectively protect metal materials used in medicine against corrosion.

## 1. Introduction

Surface modification of biomaterials is a rapidly developing area of research in many scientific centers that aim to improve the properties of materials used in medicine. The proposed techniques focus on surface modification to better meet the specific requirements of their intended applications. Metallic biomaterials, widely used in the production of implants, despite their numerous advantages, have certain limitations. For example, they are often too stiff compared to natural bone, can degrade over time, which affects their stability and durability, and are exposed to corrosion induced by the aggressive physiological environment.

Titanium and its alloys have gained particular recognition among the metallic materials used for implants. They are characterized by excellent chemical and mechanical properties, high biocompatibility, excellent corrosion resistance, low specific gravity, and modulus of elasticity [1,2,3,4,5,6,7,8,9]. To extend the life of implants, thin-layer or multilayer coatings are increasingly recommended, as these can provide protection, including effective corrosion resistance [10,11,12,13].

**Figure 1:**
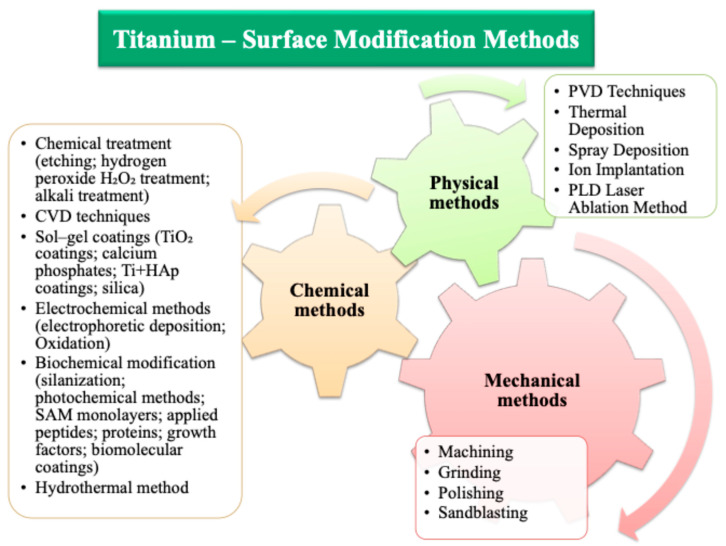
Methods of modifying the surface of titanium and its alloys—own study based on [15,20,21,22,23,24,25,26].

Changes to the surface layer lead to improved biocompatibility, corrosion resistance, and wear resistance [14]. A literature analysis shows that numerous studies confirm the benefits of surface modification of titanium and its alloys in the context of implantology [15,16,17,18,19]. Surface engineering methods can be divided into three main categories: (i) mechanical methods, (ii) chemical methods, and (iii) physical methods. Figure 1. presents methods for modifying the surface of titanium and its alloys. The figure presents various surface modification methods of titanium and its alloys, identified based on a review of numerous scientific publications. These methods aim to improve the physical, chemical, and mechanical properties and tailor its surface for specific applications, especially in the medical industry.

Sol–gel methods are widely used in medicine to produce coatings on titanium and its alloys. This leads to the creation of a new generation of biomaterials with precisely defined microstructure, chemical composition, and surface topography. Moreover, sols can be enriched with substrates that impart desired properties to the coating, such as biocompatibility or antithrombogenicity.

In this study, the production of multilayer coatings consisting of four layers, applied from two solutions—based on hydroxyapatite and vinyltrimethoxysilane—is proposed. The purpose of using hydroxyapatite coatings on metal biomaterials is to limit the release of metal ions from the substrate and to increase the bioactivity of bone tissue [27,28]. Despite the numerous advantages associated with the use of hydroxyapatite-based coatings in implantology, there are also limitations, such as their high brittleness and low fracture resistance [29].

The use of vinyltrimethoxysilane as a silicon-based coupling agent is intended to improve the performance of hydroxyapatite coatings. As part of this work, a detailed analysis of the structure and performance of VTMS/HAp/VTMS/VTMS coatings was produced and performed, including tests of thickness, adhesion of coatings to the substrate, microstructure, chemical composition, geometric structure, and corrosion resistance. In the literature, many studies address surface modifications of implants; however, to date, very few studies have investigated the use of silane-based coatings with the addition of hydroxyapatite (HAp) as an innovative material solution. The coating we propose is a new and unique approach in this field, combining the properties of silane and HAp, which can significantly improve osseointegration and implant durability. Therefore, highlighting this novel coating in the context of existing surface modification methods is of scientific importance. The results of our study confirm the excellent performance characteristics of this coating, making a valuable contribution to the development of technologies used in implantology and opening up new possibilities for clinical practice.

## 2. Materials and Methods

In this work, titanium Grade 2 was selected as the substrate for the produced coatings with the following chemical composition: Fe—max. 0.3; O—max. 0.25; C—max. 0.08; N—max. 0.03; H—max. 0.015, Ti—the rest. Samples for corrosion tests were in the form of cylinders with a diameter of 5 mm, which were mounted in polymethyl methacrylate frames using epoxy resin. For the remaining tests, rods with a diameter of 25 mm and a height of about 5 mm were used. The coating procedure was as follows: (i) wet mechanical polishing of the samples using 2000 grit sandpaper, (ii) washing of the samples with distilled water, (iii) degreasing of the samples with acetone, (iv) immersion of the samples in the prepared solutions for the appropriate time, (v) removal of excess solution using filter paper, and (vi) storage of the coated samples in a desiccator until they were completely dry. The coatings were prepared using analytically pure reagents, namely the following: (i) vinyltrimethoxysilane (VTMS) from Sigma Aldrich (St. Louis, MO, USA), (ii) hydroxyapatite powder (HAp) from Sulzer Metco (Winterthur, Switzerland), (iii) anhydrous ethyl alcohol (EtOH) from Chempur (Piekary Slaskie, Poland), and (iv) acetic acid (AcOH) from Chempur. The coatings were prepared by preparing and applying two solutions: (i) a vinyltrimethoxysilane-based solution and (ii) a hydroxyapatite-based solution. The silane-based solution was obtained by mixing vinyltrimethoxysilane, ethyl alcohol, acetic acid, and distilled water according to the procedure developed in earlier works [30,31]. The volume ratio of VTMS:EtOH:AcOH: H_2_O was 0.6:0.2:0.06:0.14. In turn, the hydroxyapatite-based solution was obtained by mixing 5 g of hydroxyapatite powder and 2.5 g of ethanol. Both solutions were mixed using a magnetic stirrer. The mixing time of the vinyltrimethoxysilane-based solution was 24–72 h, while the hydroxyapatite-based solution was 1–5 h. The produced coatings consisted of 4 layers—details regarding the formation of the coatings are included in patent application no. P.440129.

**Figure 2:**
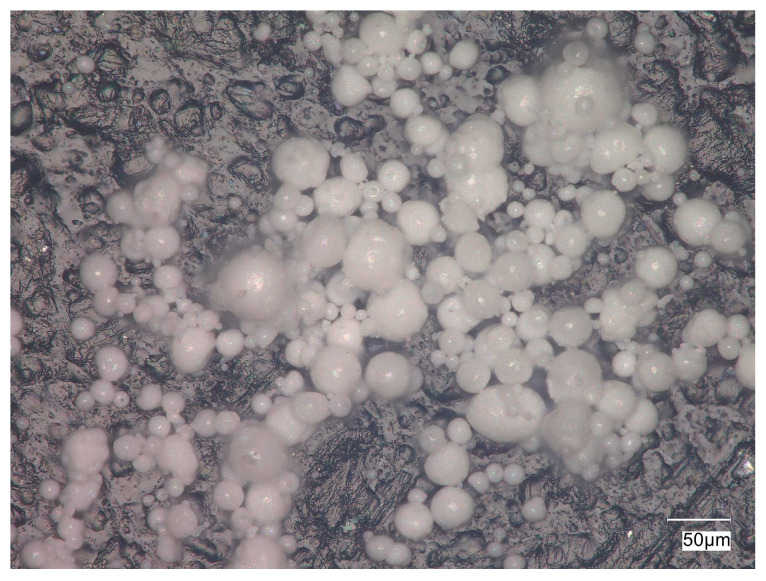
Microstructure of the hydroxyapatite powder used in the study, recorded with a KEYENCE VHX-7000 (Keyence, Mechelen, Belgium) digital microscope.

The hydroxyapatite powder used in the study has an average particle diameter of 19.93 μm, with a maximum diameter of 87.07 μm and a minimum of 5.34 μm. Figure 2 shows the microstructure of the hydroxyapatite powder, as observed using digital microscopy.

The adhesion of the coatings to the substrate was tested using Scotch^TM^ tape (Scotch^TM^ Brand, St. Paul, MN, USA) with the pull-off method according to ASTM D3359 standard [32]. The coating thickness was measured using a Testan DT-10 AN 120 157 (Anticorr, Gdansk, Poland) thickness gauge (Anticorr, Gdansk, Poland). The measurement consisted of establishing a calibration point on an uncoated substrate, measuring the coating thickness 10 times, and determining the average value of the coating thickness.

Microstructural studies were performed using a JEOL JSM-6610 LV scanning electron microscope (Jeol, Tokyo, Japan) and a KEYENCE VHX-7000 digital microscope (Keyence, Mechelen, Belgium). The chemical composition of the coatings was characterized using an EDS (Energy Dispersive Spectroscopy) X-ray microanalyzer—X-Max 20mm^2^ (Oxford Instruments, Abingdon, Oxfordshire, UK) attached to a scanning electron microscope (SEM). The structure of the produced coating was characterized using an IRAffinity-1S FTIR SHIMADZU infrared spectrophotometer (Kyoto, Japan) with a QATR 10 attachment. The roughness of the coatings was determined using a KEYENCE VHX-7000 digital microscope and a NanoScope V MultiMode 8 Bruker atomic force microscope (Bruker, Bremen, Germany), which was also used to measure the topography of the coatings. During the AFM measurements, the tapping mode was used with a scan rate of 1 Hz. The characteristics of the cantilever used in the measurements were as follows: Bruker, model—TESP; material: 0.01–0.025 Ohm-cm Antimony (n) doped Di; f: 305–368 kHz; K: 20–80 N/m.

To determine the surface roughness, both two-dimensional and three-dimensional surface analyses were taken into account. In the two-dimensional analysis of surface irregularities, the following parameters were taken into account to describe the roughness: the arithmetic mean of the roughness profile deviations (Ra), the maximum height of the roughness profile (Rz), the ten-point irregularity height (RzJIS) which is the sum of the average absolute values of the five highest-profile peaks and the average absolute values of the five lowest profile depressions measured with respect to the mean line of the elementary section of the roughness profile, the maximum height of the roughness profile peak (Rp), the maximum depth of the roughness profile depression (Rv), the average height of the roughness profile elements (Rc), the total height of the roughness profile (Rt), the root mean square of the roughness profile deviations (Rq), the roughness profile asymmetry coefficient—skewness (Rsk), the roughness profile slope coefficient—kurtosis (Rku), the average width of the roughness profile elements (RSm), and the mean square elevation of the roughness profile (Rdq).

Taking into account the three-dimensional analysis of surface irregularities, the following parameters were used to describe them: the arithmetic mean deviation of the surface from the mean surface (Sa), the maximum surface height (Sz), the standard deviation of the surface irregularities (Sq), the surface asymmetry coefficient—skewness (Ssk), the surface inclination coefficient—kurtosis (Sku), the highest surface elevation (Sp), and the depth of the lowest surface depression (Sv).

The corrosion resistance tests were performed using the CH Instruments 440A measuring station (CH Instruments, Austin, TX, USA) in a three-electrode system: (i) an auxiliary electrode—platinum, (ii) a reference electrode—a saturated calomel electrode (SCE), and (iii) the tested electrode. For each sample, a potentiodynamic curve was recorded in the potential range from −1.5 V to 3.0 V.

The prepared samples were analyzed in simulated body fluid, the composition of which is presented in Table 1.

Many solutions simulate the conditions prevailing in the human body, the so-called simulated physiological solutions, among which SBF is characterized by the largest number of components and the highest concentration of chloride ions in comparison to other solutions used, among others, in the work [31].

## 3. Results and Discussion

### 3.1. Peeling Test of VTMS/HAp/VTMS/VTMS Coating

The results showed very good adhesion of the coatings to the base material. After five times of sticking and peeling the tape from the surface, no visible damage or defects were found, which indicates strong adhesion. After each tapping and unsticking cycle, the surface was microscopically analyzed in detail.

Analysis of these results suggests that the presented coatings have an exceptional ability to maintain integrity in contact with the titanium substrate, which may be crucial in the context of their use in medical environments, where durability and reliability of corrosion protection are particularly important. Such results provide a solid basis for further development and potential implementation of these materials in medical practice, especially in the case of implants and other devices in contact with body tissues.

The pull-off test was used as a preliminary method to evaluate the adhesion of the coatings. In the future, we plan to conduct more advanced measurements to provide detailed, quantitative information on the adhesion of the coatings.

### 3.2. Thickness of the Produced Coatings

The average thickness of the VTMS/HAp/VTMS/VTMS coating deposited on a Grade 2 titanium substrate was 5.27 μm. Thickness analysis allows for the assessment of the efficiency of the coating production process and its potential functionality in medical applications. This value indicates the appropriate thickness, which provides not only effective protection against corrosion but also is optimal, allowing the coating to cooperate with the titanium substrate in dynamic physiological conditions. The thickness of the coating in this range can affect its resistance to mechanical damage and the ability to maintain chemical stability in long-term use, which is important in the context of its potential use in implantology and other medical devices.

### 3.3. Microstructural Studies of the VTMS/HAp/VTMS/VTMS Coating

**Figure 3:**
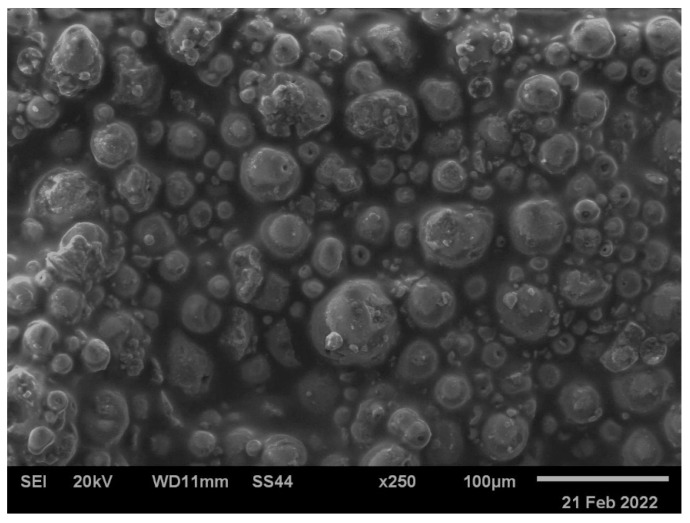
The microstructure of the VTMS/HAp/VTMS/VTMS coating deposited on Grade 2 titanium was imaged using a scanning electron microscope.

A detailed analysis of the morphology of the produced coatings was carried out. Figure 3 shows the microstructure of the VTMS/HAp/VTMS/VTMS coating deposited on Grade 2 titanium. Based on the recorded images, it was found that the coating was evenly distributed on the substrate surface, showing no signs of cracks or unevenness. Its structure was compact and uniform, and the coating thickness was uniform, without visible fluctuations.

The particles present in the coating structure, visible in the microscopic images, are related to the presence of hydroxyapatite (HAp) in powder form, which confirms the effective incorporation of this substance into the coating structure.

The morphological analysis indicates the high quality of the produced coating, which may be of significant importance in the context of its medical applications, especially in the case of implants, where homogeneity, lack of cracks, and an appropriate surface structure are key to ensuring long-term durability and effectiveness of corrosion protection. The homogeneity of the coating may also promote better integration of the material with body tissues, which is important from the point of view of biocompatibility. The high quality of the produced coating applied using the sol–gel method refers to key properties such as uniformity and thickness control of the coating, good adhesion to the substrate, and corrosion resistance, which determine their effectiveness and durability in various applications.

### 3.4. Chemical Composition of the VTMS/HAp/VTMS/VTMS Coating

**Figure 4:**
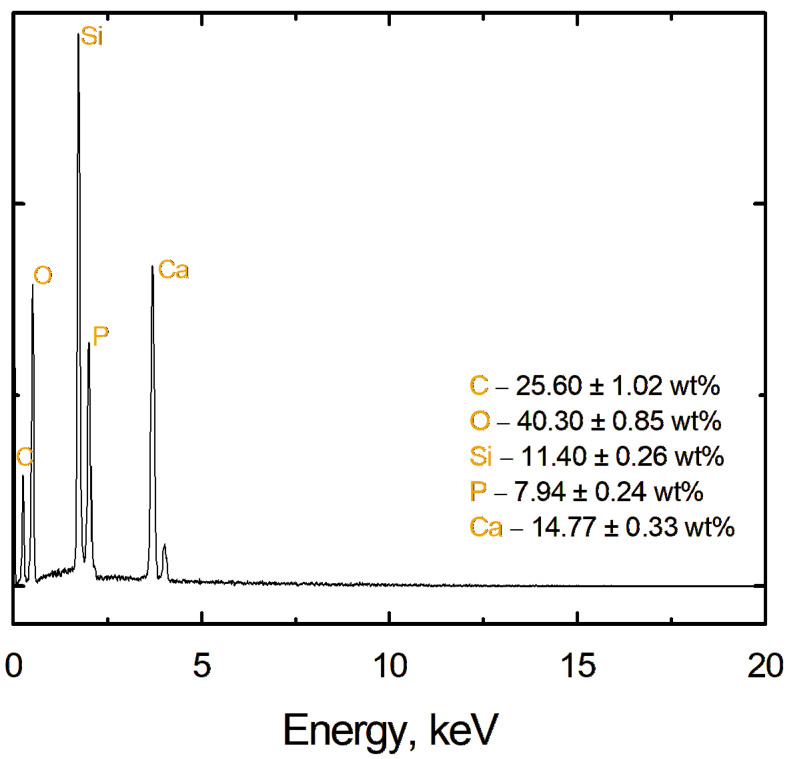
EDS spectrum recorded for the VTMS/HAp/VTMS/VTMS coating deposited on Grade 2 titanium, showing the distribution of individual elements.

A detailed analysis of the chemical composition of the multilayer coating deposited on a Grade 2 titanium substrate was carried out. As a result of this analysis, the presence of several key elements, C, O, Si, P, and Ca, was revealed, which are an integral part of the coating structure. Figure 4 shows the EDS spectrum, which allowed the identification of these elements, as well as the determination of their content in VTMS/Hap/VTMS/VTMS coating. The measurement results also include measurement uncertainties expressed in weight percent.

Analysis of the chemical composition of the coating provided important information on the structure and potential functional properties of the coatings. The EDS analysis results confirm that the coating consists of appropriate components that can ensure its durability, corrosion resistance, and beneficial biological interactions in medical applications.

### 3.5. Characteristics of the VTMS/HAp/VTMS/VTMS Coating Structure

**Figure 5:**
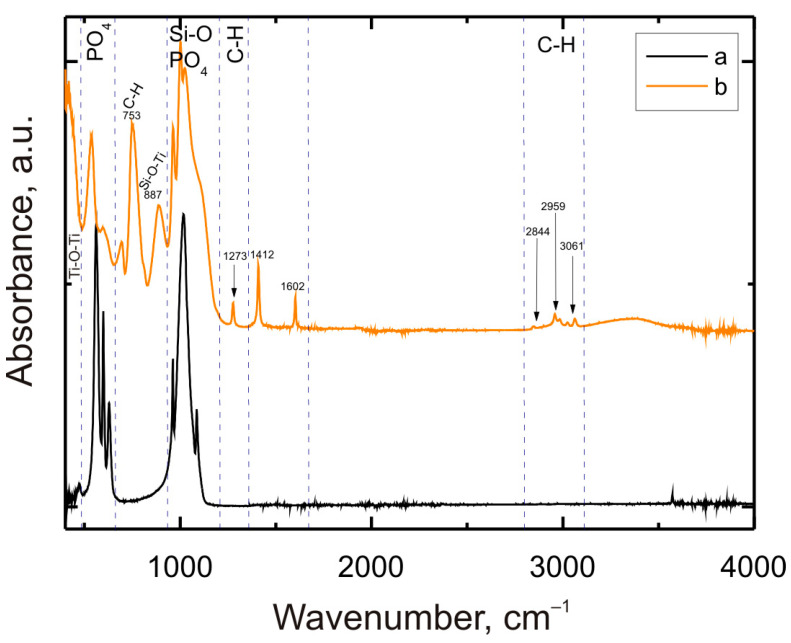
FTIR spectra were recorded for hydroxyapatite powder (a) and the VTMS/HAp/VTMS/VTMS coating deposited on Grade 2 titanium (b).

The spectrum recorded for the VTMS/HAp/VTMS/VTMS coating deposited on Grade 2 titanium, presented in Figure 5, reveals the presence of characteristic bands in different wavenumber ranges, which indicate specific functional groups included in the coating. In addition to the spectrum for the multilayer coating, a spectrum was also recorded for hydroxyapatite powder. The spectrum obtained for the VTMS/HAp/VTMS/VTMS coating shows bands for wavenumbers around 400 cm^−1^, which originate from Ti-O-Ti stretching vibrations [34]. The bands in the range of 500–660 cm^−1^ indicate the presence of the PO_4_ group in hydroxyapatite. The peak recorded for the wave number of 753 cm^−1^ is related to the C-H bond, while for the wave number of 887 cm^−1^, it most probably comes from the Si-O-Ti bond. The bands in the range of 930–1220 cm^−1^ result from both the stretching of the Si-O bond and the presence of PO_4_, which is confirmed by, among others, the spectrum recorded for the hydroxyapatite powder itself [35,36]. The peak recorded for the wave number of 1273 cm^−1^ occurs due to the bending of the C-H bond, while the peaks at 1412 and 1602 cm^−1^ are related to the unconjugated C=C bond [37]. Characteristic C-H stretching bands occur at wave numbers 2844, 2959, and 3061 cm^−1^ [35].

Based on the analysis of the spectra obtained for the VTMS-HAp-VTMS-VTMS coating, the occurrence of absorbance maxima for the functional groups Ti-O-Ti, Si-O, C-H, C=C, Si-O-Ti, PO_4_^3−^ can be observed.

The presence of characteristic bands for Ti-O-Ti, PO₄, Si-O-Ti, and C-H confirms the effective deposition of VTMS and HAp layers on the titanium surface and their mutual interactions. The obtained chemical composition suggests that the coating may exhibit bioactive properties, which is particularly important in the context of medical applications such as implants. The bioactivity of the coating, due to the presence of hydroxyapatite, can support bone integration, while the silanes contribute to the stability and durability of the coating, ensuring its resistance to mechanical and chemical factors.

### 3.6. Geometric Structure of the VTMS/HAp/VTMS/VTMS Coating

#### 3.6.1. Roughness of the Grade 2 Titanium

**Figure 6:**
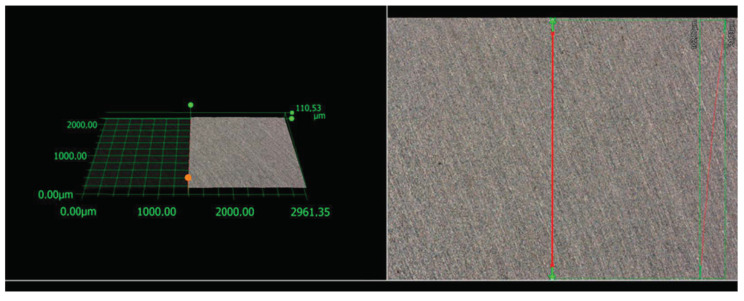
Line roughness was measured using a KEYENCE VHX-7000 digital microscope for a Grade 2 titanium.

Figure 6 shows the microstructure and surface topography of Grade 2 pure titanium obtained using a digital microscope. By capturing isometric images and control maps with high sharpness and resolution, 2D profile roughness parameters and surface roughness parameters were determined for the substrate used for the coating application. Table 2 presents the 2D profile roughness parameters, while Table 3 presents the surface roughness parameters determined for Grade 2 titanium.

#### 3.6.2. Roughness of the VTMS/HAp/VTMS/VTMS Coating Before Corrosion Tests

**Figure 7:**
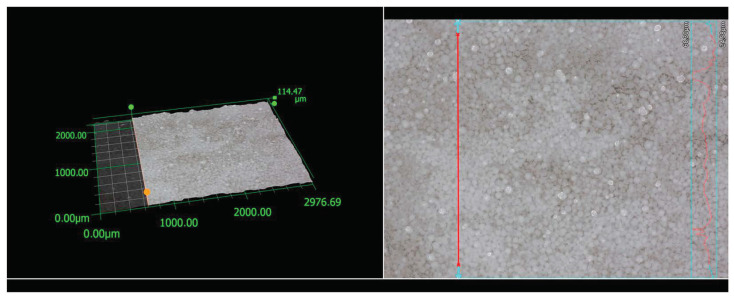
Line roughness was measured using a KEYENCE VHX-7000 digital microscope for a VTMS/HAp/VTMS/VTMS coating deposited on a Grade 2 titanium substrate.

Figure 7 presents the microstructure and surface topography of the tested coatings obtained on a digital microscope, which was performed to determine the 2D profile roughness and surface roughness parameters. The obtained isometric images and contour maps were characterized by high sharpness, contrast, and resolution. Based on the measurements performed, information on the profile, its height, and stereometric parameters was obtained.

The 2D profile roughness and surface roughness parameters obtained for the tested coatings are presented in Table 4 and Table 5.

#### 3.6.3. Roughness of the VTMS/HAp/VTMS/VTMS Coating After Corrosion Tests

**Figure 8:**
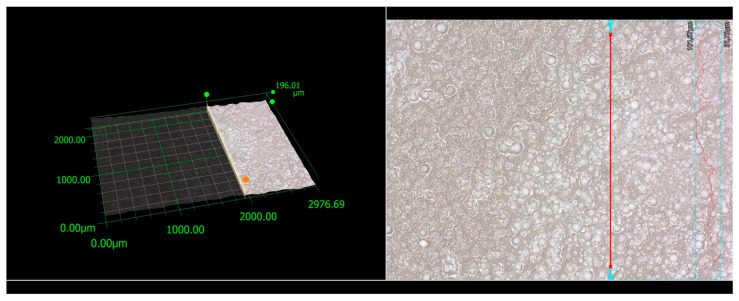
Line roughness was measured using a KEYENCE VHX-7000 digital microscope for a VTMS/HAp/VTMS/VTMS coating deposited on a Grade 2 titanium substrate after corrosion testing in simulated body fluid.

Assessment of surface topography was also performed for coatings after corrosion resistance tests. This analysis aimed to check the effect of the corrosive solution—in this case, simulated body fluid—on the geometric structure of the coating. Figure 8 presents the microstructure and surface topography of the tested coatings obtained on a digital microscope, which was performed to determine the 2D profile roughness and surface roughness parameters. Table 6 and Table 7 present the two-dimensional and three-dimensional roughness parameters for the coating after corrosion tests.

Analyzing the obtained isometric images and contour maps, it can be seen that the morphology and topography of the coatings differ from each other. Based on the analysis of the surface roughness parameters, both in the context of the profile and topography of the coatings, it can be concluded that the exposure of the coating to the corrosive solution leads to an increase in the surface roughness.

Literature data show that bioactive coatings, such as hydroxyapatite (HAp) or titanium anti-corrosion coatings, can reduce the corrosion rate of the implant material and, at the same time, improve osteointegration due to adequate surface roughness. Many authors have highlighted the benefits of a surface with the appropriate level of roughness for the primary stability and osseointegration process of implants, influencing both mechanical properties and cellular response. However, despite decades of research, the impact of implant surface roughness and other surface parameters on the biological response remains a complex and controversial topic. Some studies also point to opposing trends, suggesting that certain levels of roughness may negatively affect osteoblast behavior or lead to different outcomes in the osseointegration process. To date, no clear consensus has been reached on which surface parameters reliably and definitively predict osteoblast behavior on titanium and titanium alloy implant surfaces [38,39].

### 3.7. Topography of the Produced Multilayer Coatings

**Figure 9:**
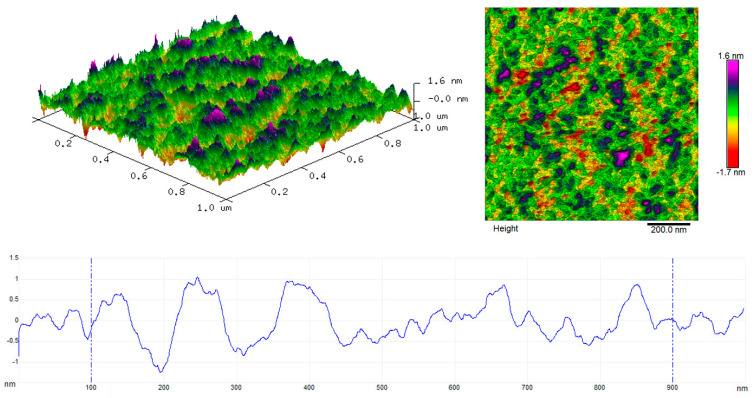
Three-dimensional and two-dimensional topography and roughness profile of the VTMS/Hap/VTMS/VTMS coating deposited on Grade 2 titanium.

Figure 9 shows the 2D and 3D topography image and the roughness profile recorded for the VTMS/Hap/VTMS/VTMS coating deposited on Grade 2 titanium. The roughness of the multilayer coatings recorded using the atomic force microscope was 0.366 nm. The differences in roughness obtained using the atomic force microscope and the digital microscope result from the analysis of surfaces of different sizes. The area examined with an atomic force microscope was only 1 × 1 μm, which may affect the accuracy and detail of the measurements obtained.

### 3.8. Corrosion Resistance Tests

**Figure 10:**
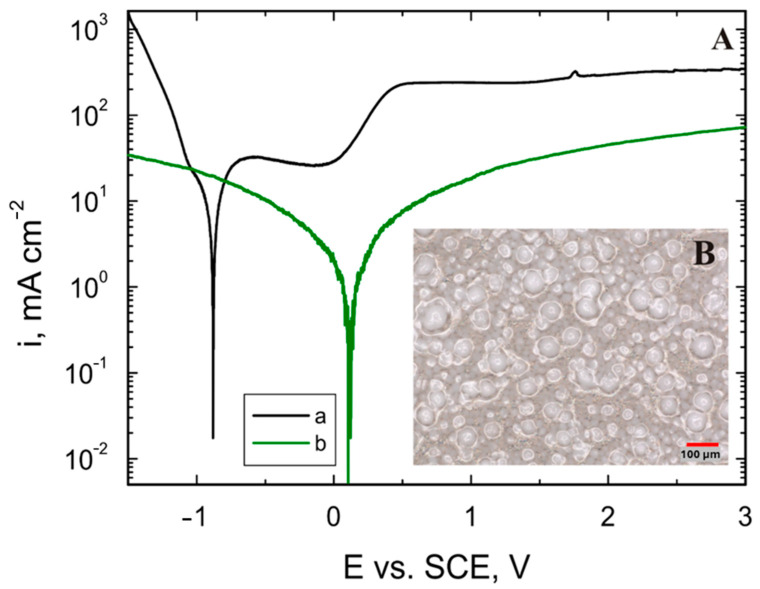
Potentiodynamic polarization curves (**A**) were analyzed in simulated body fluid for (a) Grade 2 titanium and (b) the coating deposited on Grade 2 titanium, along with the coating microstructure after corrosion tests in simulated body fluid (**B**).

Figure 10 shows the potentiodynamic polarization curves recorded for (a) Grade 2 titanium and (b) the VTMS/HAp/VTMS/VTMS coating deposited on Grade 2 titanium (A) and a photo of the coating after corrosion tests in simulated body fluid (B), which confirms the lack of coating damage as a result of corrosion resistance tests.

Based on the recorded potentiodynamic polarization curves, the polarization resistances were determined for a clean Grade 2 titanium substrate (a) and a VTMS/HAp/VTMS/VTMS coating deposited on Grade 2 titanium (b). Potentiodynamic measurements were performed with log(i) = f(E), and the curves were recorded at a polarization rate of 0.01 V/s. The relatively high scan rate may have limited the depth of alloy and coating etching during a single measurement. However, it was sufficient to record Faradaic processes on the electrode. The total duration of a single measurement was 7.5 min. In the future, we will also consider the use of gravimetric and impedance tests to determine precise values of corrosion rates.

**Figure 11:**
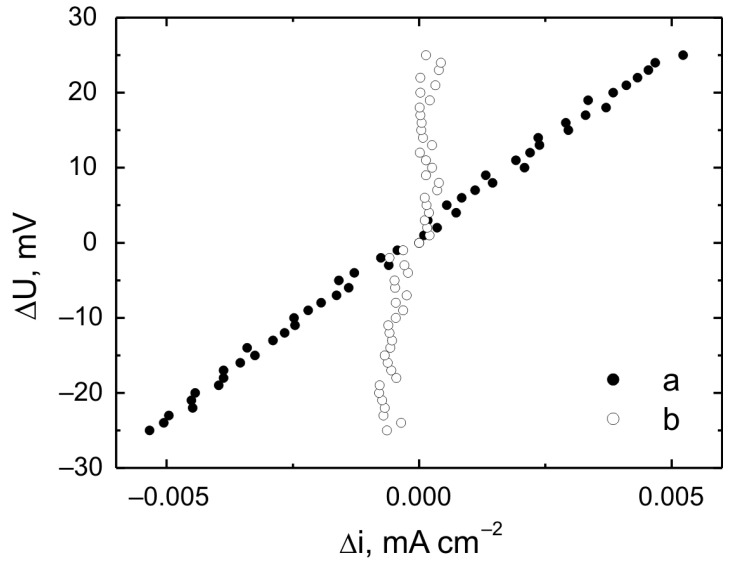
Linear polarization resistance plots were recorded for (a) Grade 2 titanium and (b) the coating deposited on Grade 2 titanium in simulated body fluid.

Figure 11 shows linear graphs of the polarization resistance for Grade 2 titanium and a VTMS/HAp/VTMS/VTMS coating deposited on Grade 2 titanium. Linear polarization resistance plots show the dependence ΔU = f(Δi) near E_corr_. For potentials slightly different from E_corr_ (±25 mV), the external current density is a linear function of the potential, while the slope of the corresponding lines is a measure of the polarization resistance, which confirms the Stern–Hoar equation [40]. Table 8 shows the values of the corrosion potential and polarization resistance for a clean Grade 2 titanium substrate (a) and a VTMS/HAp/VTMS/VTMS coating deposited on a Grade 2 titanium substrate (b).

As is known, the higher the value of the corrosion potential (E_corr_) and the lower the values of the anodic currents, the higher the resistance to the corrosive environment of the material.

The analysis of the presented potentiodynamic curves and polarization resistance measurements in the simulated body fluid show that the best results were achieved for the titanium alloy covered with the VTMS-HAp-VTMS-VTMS coating (Figure 10b). The use of this coating on the titanium surface increases the resistance of the tested material to corrosion, providing effective anti-corrosion protection. The VTMS-HAp-VTMS-VTMS coating on the titanium substrate significantly reduced both the cathodic and anodic current densities compared to the material without the coating. Additionally, a significant shift in the corrosion potential (E_corr_) towards positive values was observed, which is illustrated by the change from E_corr_ = −0.88 V for titanium without the coating to E_corr_ = 0.10 V for the Grade 2 titanium with the VTMS-HAp-VTMS-VTMS coating. The highest polarization resistance value was obtained for the VTMS-HAp-VTMS-VTMS coating, which confirms high corrosion resistance.

Silane-based sol–gel coatings can be used to protect metals and alloys as an alternative to toxic chromate conversion coatings [41]. Silane coatings can act as primers by creating a physical barrier that restricts the access of corrosive agents to the metal surface, thus protecting against corrosion. The effectiveness of silane coatings relies on the formation of a defect-free layer with strong adhesion. Several factors influence the formation of silane coatings, including curing conditions, solution pH, surface preparation, coating thickness, application method, and particle size matrix [42,43,44].

## 4. Conclusions

High potential of the new material solution: The results of the conducted tests confirm the high potential of the proposed material solution, which can provide effective corrosion protection for metallic materials used in medical applications, especially in implantology.

Effectiveness of the applied coating: The VTMS-HAp-VTMS-VTMS multilayer coating, produced by the sol–gel method using VTMS silane and hydroxyapatite (HAp) powder, demonstrates excellent adhesion to the substrate and effectively protects the titanium substrate from the corrosive environment. Its thickness is approximately 5.27 μm, and its roughness values are Ra = 5.74 μm and Sa = 11.35 μm, which may promote better integration of the implant into bone tissue.

Bioactivity of the coating: Spectral analysis of the coating revealed the presence of characteristic bands for the functional groups Ti-O-Ti, Si-O, C-H, C=C, Si-O-Ti, and PO₄³-. The presence of these chemical groups suggests that the coating exhibits bioactive properties, which is particularly important for medical applications. The hydroxyapatite in the coating may promote the osteointegration process, while the silane components ensure its stability.

Improved corrosion resistance: The application of the VTMS-HAp-VTMS-VTMS coating on the titanium surface significantly improved the corrosion resistance of the tested material. These effects were evident in the shift of the corrosion potential toward more positive values, the reduction in cathodic and anodic current density, and the increase in polarization resistance, confirming the effectiveness of the applied technology in corrosion protection.

Innovation of the technology used: The use of sol–gel technology to produce VTMS/HAp/VTMS/VTMS coatings, based on vinyltrimethoxysilane (VTMS) and hydroxyapatite (HAp), represents an innovative approach in biomaterials engineering, opening up new possibilities for the production of implant materials. This method, which has not been previously described in the literature, demonstrates high efficiency and offers significant potential for further research and applications.

## 5. Patents

Patent application no. P.440129.

## Figures and Tables

**Table 1:** Chemical composition of simulated body fluid [33].

SBF, Simulated Body Fluid	
**NaCl**	8.035 g dm^−3^
**KCl**	0.225 g dm^−3^
**CaCl_2_**	0.292 g dm^−3^
**NaHCO_3_**	0.355 g dm^−3^
**MgCl_2_·6H_2_O**	0.311 g dm^−3^
**K_2_HPO_4_·3H_2_O**	0.231 g dm^−3^
**Na_2_SO_4_**	0.072 g dm^−3^
**((HOCH_2_)_3_CNH_2_)**	6.118 g dm^−3^
**HCl (1 mol dm^−3^)**	39 mL dm^−3^

**Table 2:** Two-dimensional profile roughness parameters for a Grade 2 titanium.

Ti Grade 2	
**Ra [μm]**	17.74
**Rz [μm]**	70.82
**RzJIS [μm]**	0
**Rp [μm]**	32.69
**Rv [μm]**	38.12
**Rt [μm]**	70.86
**Rq [μm]**	20.59
**Rsk**	−0.16
**Rku**	1.84
**Rdq**	0.04

**Table 3:** Surface roughness parameters for a Grade 2 titanium.

Ti Grade 2	
**Sa [μm]**	21.07
**Sz [μm]**	108.67
**Sq [μm]**	24.64
**Ssk**	−0.14
**Sku**	1.99
**Sp [μm]**	47.23
**Sv [μm]**	61.43

**Table 4:** Two-dimensional profile roughness parameters for a VTMS/HAp/VTMS/VTMS coating deposited on a Grade 2 titanium substrate.

VTMS/HAp/VTMS/VTMS Coating	
**Ra [μm]**	5.74
**Rz [μm]**	43.72
**RzJIS [μm]**	17.19
**Rp [μm]**	26.84
**Rv [μm]**	16.88
**Rc [μm]**	23.35
**Rt [μm]**	43.72
**Rq [μm]**	7.98
**Rsk**	0.94
**Rku**	5.09
**RSm [μm]**	693.91
**Rdq**	0.31

**Table 5:** Surface roughness parameters for VTMS/HAp/VTMS/VTMS coating deposited on a Grade 2 titanium substrate.

VTMS/HAp/VTMS/VTMS Coating	
**Sa [μm]**	11.35
**Sz [μm]**	112.68
**Sq [μm]**	14.74
**Ssk**	0.02
**Sku**	3.58
**Sp [μm]**	68.88
**Sv [μm]**	43.8

**Table 6:** Two-dimensional profile roughness parameters for a VTMS/HAp/VTMS/VTMS coating deposited on a Grade 2 titanium substrate after corrosion testing in simulated body fluid.

VTMS/HAp/VTMS/VTMS Coating After Corrosion Tests	
**Ra [μm]**	12.22
**Rz [μm]**	49.57
**RzJIS [μm]**	29.86
**Rp [μm]**	20.19
**Rv [μm]**	29.38
**Rc [μm]**	29.86
**Rt [μm]**	49.57
**Rq [μm]**	13.57
**Rsk**	−0.07
**Rku**	1.85
**RSm [μm]**	548.55
**Rdq**	0.16

**Table 7:** Surface roughness parameters for VTMS/HAp/VTMS/VTMS coating deposited on a Grade 2 titanium substrate after corrosion testing in simulated body fluid.

VTMS/HAp/VTMS/VTMS Coating After Corrosion Tests	
**Sa [μm]**	26.86
**Sz [μm]**	190.19
**Sq [μm]**	33.56
**Ssk**	−0.03
**Sku**	2.72
**Sp [μm]**	99.03
**Sv [μm]**	91.16

**Table 8:** Summary of parameters obtained for individual samples.

Sample	Corrosion Potential [V]	Polarization Resistance [kΩ cm^2^]
*Ti Gr2*	*−0.88*	*4.862 ± 0.038*
*Ti Gr2 + coating*	*0.10*	*34.267 ± 2.761*

## Data Availability

The original contributions presented in this study are included in the article. Further inquiries can be directed to the corresponding authors.
